# *Helicobacter pylori* infection combined with non-alcoholic fatty liver disease increase the risk of atherosclerosis: Focus in carotid artery plaque

**DOI:** 10.1097/MD.0000000000014672

**Published:** 2019-03-01

**Authors:** Lo-Yip Yu, Kuang-Chun Hu, Chun-Jen Liu, Chung-Lieh Hung, Ming-Jong Bair, Ming-Jen Chen, Horng-Yuan Wang, Ming-Shiang Wu, Shou-Chuan Shih, Chuan-Chuan Liu

**Affiliations:** a Division of Gastroenterology, Department of Internal Medicine, Healthy Evaluation Center; b Division of Cardiology, Department of Internal Medicine, Mackay Memorial Hospital, Taipei; c Division of Gastroenterology, Department of Internal Medicine, Mackay Memorial Hospital, Taitung Branch, Taitung; d Graduate Institute of Clinical Medicine, National Taiwan University College of Medicine, Taipei, Taiwan.

**Keywords:** carotid artery plaque, helicobacter pylori infection, non-alcoholic fatty liver disease

## Abstract

Atherosclerosis has severe consequences on human health. Carotid artery plaques are a condition typically caused by atherosclerosis. Previous studies showed that nonalcoholic fatty liver disease (NAFLD) and *Helicobacter pylori* (*H pylori*) are risks factors for carotid artery plaque formation. We hypothesize that the combination of NAFLD with *H pylori* infection increases the risk of carotid artery plaque formation.

A total of 4669 subjects aged > 40 years who underwent routine health checkups between January 2006 and December 2015 were retrospectively reviewed. A serial examination, including abdominal ultrasound, carotid artery ultrasound and esophago-gastroduodenoscopy (EGD), and biopsy urease testing, was conducted.

In total, 2402 subjects were enrolled. There were no differences in *H pylori* infection status among patients with or without NAFLD. There was a trend of more participants with both NAFLD and *H pylori* infection (number [N]=583) presenting carotid artery plaque (N = 187,32.08%) than participants without NAFLD and *H pylori* infection (N = 589) who presented plaque formation (N = 106, 18.00%). Participants who had both *H pylori* infection and NAFLD had the highest risk of any carotid artery plaque (odds ratio [OR], 1.93; 95% confidence interval [CI], 1.413–2.636) based on a multivariate logistic regression analysis. This analysis also showed that age >60 years, male sex, low-density lipoprotein (LDL) >130 mg/dL, and *H pylori* infection were independent risk factors for concomitant NAFLD and carotid artery plaque formation.

The combination of *H pylori* infection and NAFLD increases carotid artery plaque formation. *H pylori* eradication and NAFLD control may be warranted to prevent carotid artery plaque formation.

## 1 Introduction

Atherosclerosis, a pathological plaque formation within blood vessels that initiates thickening of the intima (the earliest lesion in the arterial wall), hardening of the arteries, and narrowing of the lumen, is the main trigger of overall cardiovascular disease (CVD) or cerebrovascular accident (CVA).^[1]^ To study atherosclerosis, a noninvasive predictor that measures carotid intima-media thickness (IMT) is commonly used. Increased IMT is associated with the presence and severity of atherosclerosis.^[2]^ Interventional trials have used changes in the IMT as a surrogate measure of therapy outcomes.^[3]^ The pathogenesis of atherosclerosis involves vascular inflammation, injury response, degeneration, and thrombosis.^[4]^ Risk factors in the formation, progression, and destabilization of atherosclerotic plaques include hypertension, hyperlipidemia, diabetes mellitus (DM), smoking habits, sex, and family history.^[5]^ Nevertheless, no condition or exposure, either individually or in combination, completely explains the occurrence and progression of atherosclerosis, as other factors are likely to be involved.^[6]^

Nonalcoholic fatty liver disease (NAFLD) is defined by an excessive accumulation of fat in the liver parenchyma of patients who consume little of no alcohol, and it is the most common cause of chronic liver disease in Western countries. The NAFLD includes a wide spectrum of liver diseases ranging from steatosis alone, which is usually a benign and nonprogressive condition, to nonalcoholic steatohepatitis (NASH), which may progress to liver fibrosis and cirrhosis.^[7]^ A growing body of evidence suggests that NAFLD itself might contribute directly to a higher risk of atherosclerosis independently of other potential confounding factors such as obesity, hypertension, hyperlipidemia and DM.^[8]^ The underlying biological mechanisms linking NAFLD and atherosclerosis include chronic inflammation, dyslipidemia, increased hepatic insulin resistance, oxidative stress and decreased adiponectin concentrations.^[9–11]^

Over the past decade, awareness of the possible associations between atherosclerosis and certain infectious pathogens has steadily increased.^[12]^ The Gram-negative curved bacillus *Helicobacter pylori* (*H pylori*) colonizes the gastrointestinal system in approximately half of all adults and is more common in developing countries than in Western countries. It is associated with chronic gastritis, peptic ulcer disease, and even gastric cancer.^[13–16]^ Accumulating evidence has found a relationship between *H pylori* infection and extragastrointestinal diseases, including liver disease, CVD, obesity, and type 2 DM. Previous studies reported that *H pylori* is present in human carotid atherosclerotic lesions. The infectious process within the vessel wall may be responsible for the initiation, progression, and complication of atherosclerotic plaque formation.^[17]^ Chronic infection with *H pylori* has been seroepidemiologically linked to atherosclerosis via indirect effects, such as systemic inflammation or autoimmune reactions.^[18]^

In this retrospective study, we enrolled a large sample of apparently healthy people who had undergone routine checkups. We hypothesize that the combination of NAFLD and *H pylori* infection increases the risk of carotid artery plaque formation due to chronic inflammation. We investigated the core risk factors for synchronous NAFLD and carotid artery plaque formation.

## 2 Materials and methods

### 2.1 Patient selection

We retrospectively analyzed the data of 4669 subjects who had undergone routine checkups at MacKay Memorial Hospital, Taipei, Taiwan, between January 2006 and December 2015. Asymptomatic individuals who had undergone esophago-gastroduodenoscopy (EGD) on the same day as part of a health checkup were enrolled for analysis. Carotid artery ultrasound was arranged on the same day. The inclusion criteria were as follows: aged > 40 years, underwent screening EGD for the detection of *H pylori* infection status by urease test, and abdominal ultrasound and carotid artery ultrasound examinations. We excluded patients and exclusion criteria are as follows:

1. who had previously proven acute myocardial infarction or stroke
2. were incapacitated or could not undergo carotid artery ultrasound examination
3. lacked data regarding *H pylori* urease test examination of gastric biopsy specimens or basic blood tests samples
4. had an alcoholic drinking habit or other secondary fat accumulation causes including steatogenic medication or hereditary disorders
5. had a history of liver cirrhosis, or
6. were positive for hepatitis B surface antigen or hepatitis C antibodies. After excluding 2267 subjects, a total of 2402 study participants (1602 males and 800 females) were enrolled for further study.

### 2.2 Scanning protocol and definition of NAFLD in abdominal ultrasound

The definition of NAFLD requires that firstly, there is evidence of hepatic steatosis, either by imaging or histology and secondly, there are no causes of secondary hepatic fat accumulation, such as significant alcohol consumption, use of steatogenic medication or hereditary disorders.

An experienced gastroenterologist blinded to the study aims performed the abdominal ultrasound using an HD-15 ultrasound system (Philips Medical Systems, Cleveland, OH). Participants were placed in the supine position, and both arms were raised above the head when the images were captured. Fatty liver diagnosis was made by ultrasound based on standard criteria, including parenchyma brightness, liver-to-kidney contrast, deep beam attenuation and bright vessel walls. Since we had already excluded fatty liver due to secondary fat accumulation, including alcohol consumption, patients with fatty liver were considered to have NAFLD.

### 2.3 Scanning protocol and definition of carotid artery lesion in ultrasound examination

Ultrasonography of the common carotid artery, carotid bifurcation and internal carotid artery of the left and right carotid arteries was performed using a 7.5-MHz linear-array transducer (ATL Ultra-Mark IV). On a longitudinal, two-dimensional ultrasound image of the carotid artery, the anterior (near) and posterior (far) walls of the carotid artery were displayed as 2 bright white lines separated by a hypoechogenic space. All participants were examined by experienced ultrasonographers. The images were stored using the cine function during the electrocardiographic R-wave in the cardiac cycle. Still images of the arterial wall were saved, and measurements were subsequently performed on the stored digital images. Analyses were performed using the AMS program package. A plaque was defined as a distinct area with an IMT > 50% greater than that at neighboring sites.

### 2.4 Scanning protocol and definition of *H pylori* infection

*H pylori* infection was detected with a biopsy urease test (CLO test, Pronto Dry, Gastrex, Poland) using standard EGD with gastrofiberscopes (GIFQ260, Olympus Optical, Tokyo Japan). A specimen for biopsy urease testing of each subject was taken from the gastric antrum using biopsy forceps and assessed within 60 minutes. The agar color turned from yellow to red when the biopsy specimen was infected by *H pylori*, which expresses an intracytoplasmic urease.

### 2.5 Clinical data collection and questionnaire

Baseline characteristics (age, gender, height, weight, body mass index, blood pressure, personal medical history and current medicine use, family history of 1st-degree relatives and smoking) were obtained from a questionnaire completed at the time of the physical checkups.

Clinical data included levels of fasting plasma glucose AC, hemoglobin A1C (HbA1c), triglyceride (TG), low-density lipoprotein (LDL), complete blood count (CBC), and high-sensitivity C-reactive protein (hs-CRP), all of which were obtained from the participants on the same health checkup day as when the EGDs and abdominal sonography were performed. Carotid artery ultrasound examination data were collected on the same day or within 12 months when the participants underwent annual physical checkups.

This study was approved by the MacKay Memorial Hospital Institutional Review Board (12MMHIS163).

### 2.6 Statistical analysis

The following variables were recorded for each subject: age, sex, body mass index (BMI), HbA1c, lipid levels, smoking status, and *H pylori* status. When the data for continuous variables fit a normal Gaussian distribution, a *t* test was applied to compare *H pylori*-positive and *H pylori*-negative participants, and the data are expressed as the mean ± standard deviation (SD). Categorical variables are expressed as numbers (percentage). Unadjusted odds ratios (ORs) with 95% confidence intervals (CIs) were computed for potential predictors of carotid artery plaque formation. Multivariate logistic regression analysis was applied to compute the adjusted OR (95% +-9 for predictors of carotid artery plaque formation. All variables with a *P* < .2 on the univariate analysis were selected for multivariate logistic regression. The final model was developed using a stepwise backward approach. All variables with *P* < .05 were considered statistically significant and remained in the final model. All analyses were performed using SAS version 9.1 (SAS Institute, Cary. NC).

## 3 Results

### 3.1 Patient characteristics

Table 1 shows the demographic data of patients with and without NAFLD. There were significant differences in all variables except age, use of antiplatelet agents and *H pylori* infection status.

**Table 1:**
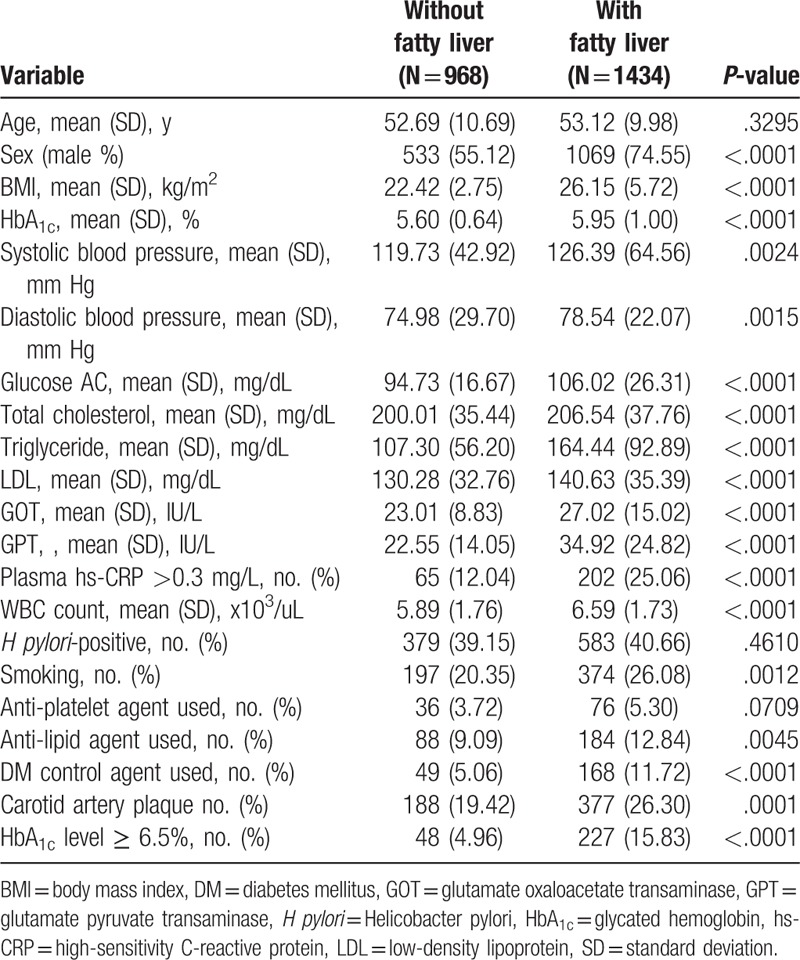
Demonstrate participants with and without fatty liver.

Table 2 shows the demographic data of patients with NAFLD either with or without *H pylori* infection. Only four variables showed significant differences: total cholesterol, white blood cell (WBC) count, use of DM agents and carotid artery plaque status.

**Table 2:**
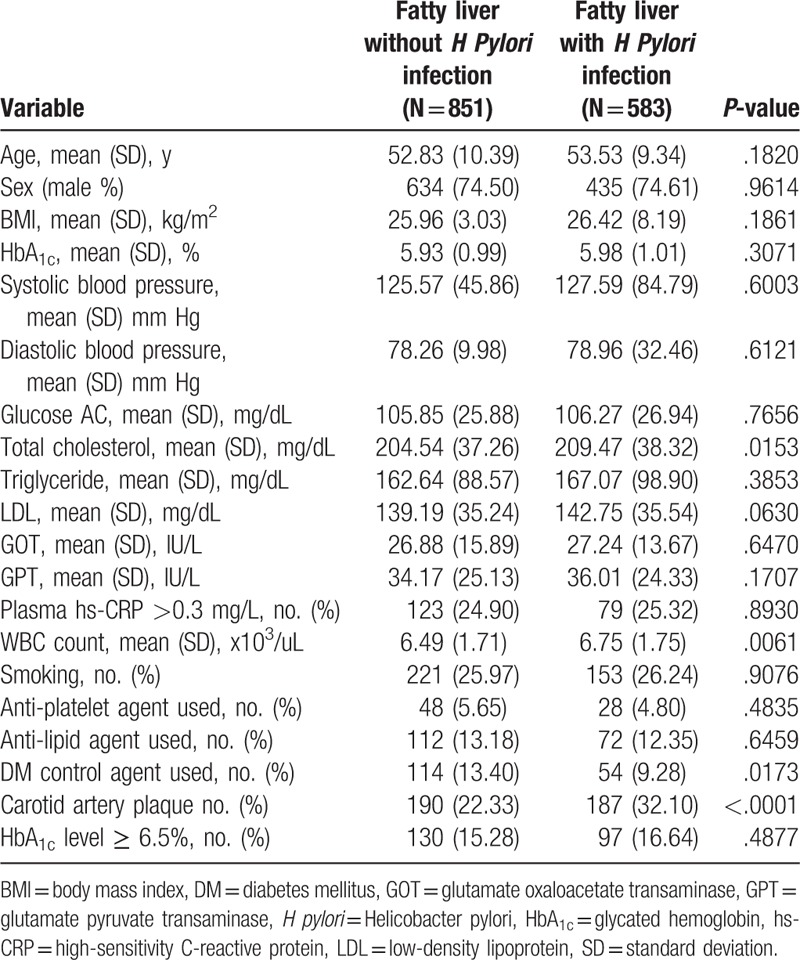
Demonstrate participants with fatty liver with and without *H pylori* infection.

### 3.2 Risk of any carotid artery plaque according to NAFLD and *H pylori* infection status

Table 3 shows that of the total subset of participants without NAFLD and *H pylori* infection (N = 589), 106 (18.00%) suffered from carotid artery plaque, a total of 82 of 379 participants (21.64%) with *H pylori* infection suffered from only carotid artery plaque and 190 of 851 participants (22.33%) with NAFLD had only carotid artery plaque. However, we observed a trend that a higher proportion of participants with both NAFLD and *H pylori* infection presented carotid artery plaque (187 of 583 participants, 32.08%).

**Table 3:**
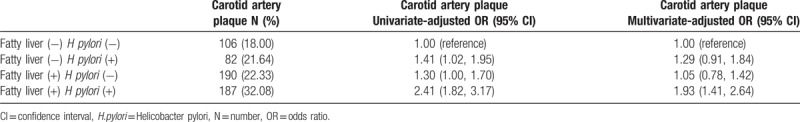
The risk of any carotid artery plaque according to NAFLD and *H pylori* infection.

### 3.3 Univariate analysis and multivariate logistic regression for predictors of any carotid artery plaque

We evaluated the impact of *H pylori* infection and NAFLD on the risk of carotid artery plaque in this study. We classified participants based on *H pylori* infection status and NAFLD status. The risk of any carotid artery plaque was higher in participants who had *H pylori* infection or NAFLD than in those without either condition (Table 3). Participants with *H pylori* infection and NAFLD had the highest risk of any carotid artery plaque (OR, 1.93; 95% CI, 1.41–2.64) in the multivariate logistic regression. Therefore, the combination of *H pylori* infection and NAFLD increases the risk of carotid artery plaque formation.

### 3.4 Univariate analysis and multivariate logistic regression for predictors of NAFLD combined with carotid artery plaque

According to the abdominal and carotid artery ultrasound results, we divided the participants into 2 groups (Table 4): First, NAFLD combined with carotid artery plaque formation. Second, NAFLD without carotid artery plaque formation.

**Table 4:**
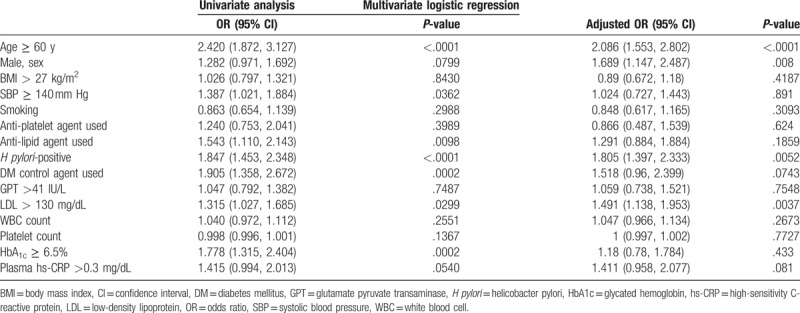
Univariate analysis and multivariate logistic regression for predictors of carotid artery plaque in patients with synchronous fatty liver and carotid plaque vs fatty liver only and no carotid plaque.

Table 4 shows the factors included in the univariate and multivariate analyses. Multivariate logistic regression analysis showed that age >60 years, male sex, LDL >130 mg/dL and *H pylori* infection were independent risk factors for concomitant NAFLD and carotid artery plaque formation. Age and sex obviously cannot be changed, but LDL and *H pylori* infection status could be treated.

## 4 Discussion

Atherosclerosis is a highly prevalent disease and is a major cause of mortality and morbidity in developed countries. When severe, it can result in coronary artery disease, CVA, peripheral artery disease or kidney problems depending on the arteries affected. Epidemiological studies have linked atherosclerosis to hypertension, hyperlipidemia, DM and smoking, but much of the risks remain unexplained.^[19]^ Our study showed that the combination of NAFLD with *H pylori* infection increases the development of carotid artery plaques, a condition typically caused by atherosclerosis.

The NAFLD is the most common chronic liver disease in Western countries, where the prevalence of NAFLD in the general population is 15 to 40%.^[20]^ In addition to having an increased risk of developing NASH, NAFLD patients are also at higher risk of cardiovascular diseases, including coronary artery disease and stroke. A meta-analysis of seven cross-sectional studies confirmed that NAFLD is strongly associated with increased carotid artery IMT and an increased prevalence of carotid atherosclerotic plaques.^[21]^ The pathogenesis linking NAFLD to atherosclerosis is not completely understood, but several pathways are plausible. The NAFLD is considered an early hepatic manifestation of metabolic syndrome in the presence of hyperglycemia, hyperinsulinemia, hyperlipidemia, and damage to vascular endothelial cells,^[22]^ all of which are associated with subclinical inflammation, a prothrombotic state, and hemodynamic alterations that may increase the risk of atherosclerosis.^[23,24]^ However, many studies have shown that NAFLD could predict an increased risk of carotid IMT independently from traditional risk factors, including insulin resistance (IR)^[25]^ and metabolic syndrome.^[26–29]^ The NAFLD is also associated with oxidative stress, which induces inflammation, destroys endothelium-dependent vasodilatation function,^[30]^ reduces the elasticity of blood vessels, promotes endothelial cell apoptosis,^[31]^ and contributes to vessel smooth muscle cell hyperplasia.^[32,33]^ The NAFLD may accelerate atherosclerosis through increased levels of atherogenic, triglyceride-rich, cholesterol-rich particles and small dense LDL particles.^[34]^ Inflammation-based mechanisms have been implicated in the pathogenesis of atherosclerosis.^[35,36]^ The association between *H pylori* infection and atherosclerosis has been reported in numerous studies in recent years. Most studies demonstrating an association between *H pylori* and atherosclerosis are serological studies, while our study assessed *H pylori* infection by the CLO test. Some mechanisms, including immune-mediated mechanisms, free radical formation, and a low-grade acute-phase response, may be the link between chronic *H pylori* infection and atherogenesis. The C-reactive protein levels, fibrinogen levels, and leukocyte count, all of which are risk factors for CVD,^[37]^ are elevated in individuals seropositive for *H pylori* and suggest a low-grade inflammatory response.^[38,39]^ Antioxidants have been shown to be decreased in subjects with *H pylori*. *H pylori* is rapidly eliminated in the circulation before it reaches the vessel wall. Some authors proposed the mechanism of immune response. *H pylori* produces 60 kDa heat shock proteins that cross react with antibodies and are a risk factor for carotid atherosclerosis.^[40]^ Interleukin-17 (IL-17) is up-regulated in the *H pylori*-colonized gastric mucosa.^[41]^ The IL-17 has been detected in human atherosclerotic lesions,^[42]^ and patients with acute myocardial infarction and unstable angina have increased peripheral IL-17 levels.^[43]^ A previous study showed that IL-17A is required for the maintenance of angiotensin II-induced hypertension and vascular dysfunction, both of which are risk factors for atherosclerosis.^[44]^ Tarantino et al suggested that IL-17, released by the visceral adipose tissue, induces eotaxin secretion via the smooth muscle cells. A strong relationship was found between chemokine eotaxin and IMT. And IL-17 is associated with early atherosclerosis in obese patients.^[45]^ However, previous studies found that *H pylori* may be present in human carotid atherosclerotic plaques and responsible for the initiation, progression, and complication of atherosclerotic plaque formation.^[46]^

Some strains of *H pylori* express the cytotoxin-associated gene-A (*cag* A), which encodes for a hydrophilic, surface-exposed protein named CagA.22. *H pylori* strains expressing CagA and carrying the *cag* pathogenicity island induce an inflammatory response in the gastric mucosa greater than that induced by strains lacking the pathogenicity island.^[47]^ In a large prospective study, Mayr et al^[48]^ provided evidence that infection with CagA-positive *H pylori* strains significantly increases the risk of early atherosclerosis in carotid arteries. Moreover, infection with CagA-positive *H pylori* strains in atherosclerotic stroke patients is associated with greater IMT and poorer short-term outcomes than those of CagA-negative patients.^[49]^ The levels of CRP, a sensitive marker for the detection of a systemic inflammatory response, trended toward higher levels in CagA-positive patients than those in CagA-negative patients.^[50]^ Molecular mimicry between CagA and proteins presented in the wall of medium- and large-sized arteries is one proposed mechanism for the initiation and development of atherosclerosis by *H pylori* infection.^[51]^

Apart from the usual risk factors resulting from NAFLD, such as sedentary lifestyle, dietary habits, obesity, hyperlipidemia, and DM, other risk factors, including *H pylori* infection, have been recently proposed. In view of its effect on metabolic variables, *H pylori* is associated with insulin resistance, which is important in the development of NAFLD.^[52]^ A randomized controlled single-blind study from Dogan et al showed that fatty liver was significantly more frequent in *H pylori*-positive patients. The extent of the fattiness as assessed by ultrasonography was also higher in the *H pylori*-positive group.^[53]^ There are some potential pathogenic mediators and mechanisms that contribute to the pathogenesis of NAFLD by *H pylori* infection, including fetuin-A, tumor necrosis factor (TNF)-α and adiponectin.^[52]^*H pylori* infection may trigger TNF-α, whereas adiponectin is secondarily increased to counterbalance the proinflammatory cascade. The TNF-α may be a mediator of both the direct and indirect effects of *H pylori* infection on NAFLD.^[54]^ Abenavoli et al found that after *H pylori* eradication therapy in an adult man, IR and fatty liver showed improvements.^[55]^

To the best of our knowledge, this report is the 1st of a positive correlation between *H pylori* infection and NAFLD complicated with carotid artery plaque formation. One strength of our study is that it included a large sample size (2402 subjects). In our study, multivariate logistic regression analysis showed that *H pylori* infection was an independent risk factor for concomitant fatty liver and carotid artery plaque. For individuals with NAFLD, *H pylori* infection increases the risk of carotid artery plaque formation, and eradicating *H pylori* infection may have therapeutic prospects in treating NAFLD and preventing carotid artery plaque formation. However, in Table 1, the demographic data of patients with and without fatty liver showed no differences in the *H pylori* infection status. The possible reasons may be that our patients were of higher social economic status and had more awareness of their health. These individuals had previously received regular health checkups, including EGD and *H pylori* eradication if present.

Our study has several important limitations. First, this was a retrospective observational study that included only patients with relatively high incomes and higher health awareness, which may not represent the general population. Second, this was a single-center study, which may have resulted in selection bias. Third, the severity of fatty liver can be divided into mild, moderate and severe according to the ultrasound findings. Our study showed only that fatty liver and *H pylori* infection are risk factors for the formation of carotid plaques, but it did not show whether the severity of fatty liver affected the observed synergy for carotid artery plaque formation. Fourth, the severity of carotid artery plaques can be divided into minimal, mild, moderate and severe according to carotid ultrasound findings. Our study reported only the incidence of carotid artery plaques in relation to NAFLD and *H pylori* infection status but did not take into consideration the severity of the plaque (s), which is a study limitation. Because our study was conducted at the Health Checkup Evaluation Center, the patients enrolled were relatively healthy. In general, patients with a severe degree of carotid artery plaque(s) or symptomatic patients would have attended outpatient clinics and regular follow-ups and would not have presented to the Health Checkup Evaluation Center, causing selection bias. Fifth, The reasons for excluding patients with a history of acute myocardial infarction or stroke are the same as those presented in the fouth limitation, causing selection bias. Sixth, the prevalence of fatty liver was 59.7% (1434/2402) in our study, which is much higher than that reported in the literature 15 to 40%.^[19]^ A possible explanation for this discrepancy is that a greater percentage of our participants were overweight (mean BMI was 26.2 in the fatty liver group, compared with 22.4 in the non-fatty liver group), so selection bias may have occurred.

## 5 Conclusions

*H pylori* infection combined with NAFLD increases the risk of carotid artery plaque formation. Given the high prevalence of *H pylori* infection in developing countries and the growing population suffering from NAFLD or even NASH, eradicating *H pylori* infection and controlling NAFLD may be warranted to prevent cardiovascular disease.

## Author contributions

**Conceptualization:** Kuang-Chun Hu.

**Data curation:** Lo-Yip Yu, Kuang-Chun Hu, Ming-Jong Bair.

**Formal analysis:** Lo-Yip Yu, Kuang-Chun Hu, Chuan-Chuan Liu.

**Methodology:** Chun-Jen Liu, Chung-Lieh Hung, Ming-Jen Chen.

**Resources:** Horng-Yuan Wang.

**Software:** Chuan-Chuan Liu.

**Supervision:** Kuang-Chun Hu, Ming-Jong Bair, Ming-Shiang Wu, Shou-Chuan Shih, Chuan-Chuan Liu.

**Writing – original draft:** Lo-Yip Yu.

**Writing – review & editing:** Lo-Yip Yu, Kuang-Chun Hu, Horng-Yuan Wang, Ming-Shiang Wu, Chuan-Chuan Liu.

Lo-Yip Yu orcid: 0000-0003-2394-8200.
